# Endurance Trained Athletes Do Not *per se* Have Higher Hoffmann Reflexes Than Recreationally Active Controls

**DOI:** 10.3389/fphys.2021.736067

**Published:** 2021-11-12

**Authors:** Raphael Bertschinger, Louis-Solal Giboin, Markus Gruber

**Affiliations:** Human Performance Research Centre, Department of Sport Science, University of Konstanz, Konstanz, Germany

**Keywords:** H-reflex, recruitment curve, spinal plasticity, sigmoidal fit, endurance athletes, cycling

## Abstract

The impact of endurance training on spinal neural circuitries remains largely unknown. Some studies have reported higher H-reflexes in endurance trained athletes and therefore, adaptations within the Ia afferent pathways after long term endurance training have been suggested. In the present study we tested the hypothesis that cyclists (*n* = 12) demonstrate higher Hoffmann reflexes (H-reflexes) compared to recreationally active controls (*n* = 10). Notwithstanding, highly significant differences in endurance performance (VO_2peak_: 60.6 for cyclists vs. 46.3 ml/min/kg for controls (*p* < 0.001) there was no difference in the size of the SOL H-reflex between cyclists and controls (H_max_/M_max_ ratio 61.3 vs. 60.0%, respectively (*p* = 0.840). Further analyses of the H and M recruitment curves for SOL revealed a significant steeper slope of the M recruitment curve in the group of cyclists (76.2 ± 3.8° vs. 72.0 ± 4.4°, *p* = 0.046) without a difference in the H-recruitment curve (84.6 ± 3.0° vs. 85.0 ± 2.8°, *p* = 0.784) compared to the control group. Cycling is classified as an endurance sport and thus the findings of the present study do not further support the assumption that long-term aerobic training leads to a general increase of the H-reflex. Amongst methodological differences in assessing the H-reflex, the training-specific sensorimotor control of the endurance sport itself might differently affect the responsiveness of spinal motoneurons on Ia-afferent inputs.

## Introduction

There is broad scientific consensus that long term endurance training causes adaptations of the cardiovascular system (Blomqvist and Saltin, 1983; Hellsten and Nyberg, 2015) and within the exercised skeletal muscles (Egan and Zierath, 2013; Camera et al., 2016). More recently, it has been shown that other organ systems are as well subject to change as a result of chronic endurance exercise. Interestingly, evidence has been provided that central nervous system (CNS) can undergo structural modifications in specific areas of the brain (Kramer et al., 1999; Colcombe et al., 2006). However, it remains unclear how these changes occur, if they have an impact on endurance performance and how different structures of the CNS can be affected (Colcombe et al., 2004; Voss et al., 2013). Besides the lack of mechanistic studies that conclusively demonstrate the role of CNS plasticity with endurance training, the potential of chronic physical exercise to induce plastic changes in the CNS can be assumed, as it is well accepted that activity-related adaptations after skill acquisition are not only restricted to certain areas of the brain but occur throughout the complete CNS (Wolpaw, 2007). On this behalf, the spinal cord takes a central role because it acts as the interface between our environment and supraspinal motor centers (Christiansen et al., 2017).

Use-dependent plasticity in the human spinal cord can be inferred by modifications in the size of the Hoffmann reflex (H-reflex; Wolpaw and Lee, 1989; Nielsen et al., 1993; Meunier et al., 2007). However, there is evidence that neuroplastic changes in the spinal cord are task-specific and thus depend on the type of exercise (Gruber et al., 2007; Vila-Cha et al., 2012). Training types like strength training, endurance training, or balance training pose very different stimuli on the exercised muscles, potentially leading to differentiated physiological adaptations as well.

The literature on cross-sectional and longitudinal studies investigating the influence of endurance training on neuroplasticity in the spinal cord is scarce. In the few studies available, there seems to be consensus that this type of training has the potential to increase the amplitude of the H-reflex in the soleus muscle (Rochcongar et al., 1979; Perot et al., 1991; Nielsen et al., 1993; Maffiuletti et al., 2001; Vila-Cha et al., 2012). An increase in the H-reflex after endurance training has been linked either to a training-induced shift toward slow motor units, which are more easily recruitable by stimulation of the Ia afferent pathway and resulting in an increase in motoneuron pool excitability (Rochcongar et al., 1979; Perot et al., 1991; Maffiuletti et al., 2001) or a decreased presynaptic inhibition of Ia terminals, resulting as well in an enhanced motoneuron responsiveness across the whole motoneuron pool (Nielsen et al., 1993; Vila-Cha et al., 2012).

Nevertheless, precipitous conclusions that endurance training *per se* leads to an increased H-reflex amplitude in the soleus is aggravated by several methodological issues. Some of the cross sectional studies lack a quantifiable independent variable to control for differences in aerobic capacity (e.g., peak oxygen consumption) between groups (Rochcongar et al., 1979; Maffiuletti et al., 2001) and included participants from a variety of different endurance sports (Rochcongar et al., 1979; Nielsen et al., 1993; Maffiuletti et al., 2001). The longitudinal studies differed in study design and H-reflex methodology. Vila-Cha et al. (2012) conducted 3 weeks of training (cycling vs. strength) and measured H-reflexes at 10% MVC force level, whereas Behrens et al. (2015) measured H-reflexes during MVCs in a training (cycling) and control group before and after an 8 week period. Thus, different outcomes in studies, for example no changes in the soleus H-reflex (Behrens et al., 2015) vs. increased soleus H-reflexes after cycling exercise (Vila-Cha et al., 2012) might be mainly attributed to differences in the experimental design. Nevertheless, based on the results from the cross-sectional studies on endurance athletes, long-term cycling training can be expected to result in general increases in soleus H-reflex excitability. Therefore, in the present study, we tested a group of cyclists, specifically trained in one endurance sport and compared them to a group of recreationally active controls, that were not specifically trained in any sport and did not perform any endurance exercise.

Furthermore, we sought to analyze the slope parameters of the H and M recruitment curves, as they can possibly gain deeper insights into motor recruitment characteristics (Funase et al., 1994; Pierrot-Deseilligny and Mazevet, 2000), and have been shown to be possibly affected by endurance training (Piscione et al., 2012).

Therefore, our aim was to test the hypotheses that (a) maximal H-reflex size of the soleus muscle is higher and (b) the slope of the H-reflex recruitment curve is steeper in a specifically trained group of endurance athletes compared to a control group. Furthermore, to demonstrate clear differences in aerobic fitness between these groups, we tested subjects for a well-established measure of aerobic capacity.

## Materials and Methods

### Subjects

Twelve male endurance-trained road cyclists (age: 25 ± 4 years, height: 183 ± 7 cm, weight: 77 ± 9 kg) were selected for the experimental group (cyclists). Two of the following three criteria had to be met to be included in the study group: A peak oxygen uptake above 60 ml/min/kg, a history of structured cycling training of more than 1 year, an annual training volume of more than 10,000 km (∼7 h weekly training) for more than 1 year.

As a control group (control) we selected 10 recreationally active males that were matched for body composition. Subjects exercised in strength training, climbing, and ball sports (age: 26 ± 6 years, height: 177 ± 6 cm, weight: 69 ± 7 kg). Subjects were included in the control group if they had no history in any endurance sport and met one of the following two criteria: recreationally active on a maximum of two occasions per week or had a peak oxygen uptake below 50 ml/min/kg. Subjects were excluded from the control group, if they exercised in any kind of endurance sport.

General exclusion criteria for this study were any history of cardiorespiratory or neuromuscular disease as well as smoking. Subjects were instructed to refrain from alcohol and caffeine 24 h before the experiments as the prior substance can increase spinal excitability (Walton et al., 2003). Furthermore, all subjects were asked to avoid any exhaustive physical activity or competition 48 h preceding participation in the study. All participants gave their written informed consent. The study was approved by the Ethics Committee of the University Konstanz (14/2019) and conducted in accordance with the latest revision of the Declaration of Helsinki.

### Experimental Procedure

All examinations were conducted in one session. First, the full recruitment curve for the SOL muscle was obtained at rest in a supine position. Skinfold measurements to assess body fat percentage and a cardiorespiratory exercise test on a cycle ergometer to obtain peak oxygen consumption and other spiroergometric variables of interest were conducted in the final part of the study.

### Surface Electromyography

Bipolar surface electrodes (Bagnoli DE-2.1, Delsys Inc., Natick, MA, United States) with an inter-electrode distance of 10 mm and an electrode size of 1x10 mm were applied according to SENIAM guidelines (Hermens et al., 1999). The electrodes were applied on SOL to obtain the recruitment curve for this muscle and on TA to control for coactivity. The position and orientation of the electrodes were consistent with the SENIAM guidelines (Hermens et al., 1999). To reduce impedance at the skin-electrode contact point we lightly abraded the skin with emery paper and cleaned it with alcohol. The reference electrode was placed on the right acromion. The EMG signal was amplified (×1,000 or ×100 if the signal saturated at ± 5V with an amplification of 1,000), bandpass filtered between 20 and 450 Hz, sampled at 4 kHz (Micro 1401, Cambridge Electronic Design Limited, Cambridge, United Kingdom) and stored on a computer.

### Electrical Nerve Stimulation

Single rectangular electrical pulses of 1 ms duration *via* custom-built surface electrodes (cathode: 5 cm^2^, anode: 24 cm^2^) were delivered to the peripheral nerve by a constant current stimulator (DS7A, Digitimer, Hertfordshire, United Kingdom). The effective current output from the stimulator was registered by a custom-built digital ammeter and stored on the computer with Signal (v5.0.8, Cambridge Electronic Design Limited, Cambridge, England) for *a posteriori* analysis.

For the recruitment curves from the right SOL muscle subjects were tested in a supine position. Subjects lay relaxed on an examination couch with their left leg fully extended and the right leg bent in a 90° knee angle. To keep the knee angle stable during the stimulation protocol, the right foot was fixed with a non-compliant strap to the couch. A semi-elastic band fixed between the knee pit and the ceiling kept the knee from tilting to either side. The cathode was placed over the posterior tibial nerve in the popliteal fossa. The anode was placed on the anterior surface of the knee. Optimal cathode position was identified by carefully moving the electrode until an optimal response in terms of a clear and pronounced biphasic M-wave signal was observed at a submaximal stimulus intensity.

Subjects were familiarized to the electrical stimulations, starting at very low intensities of ∼5 mA with rest intervals of ∼20 s. Then we identified the stimulation intensities for H-wave threshold and M_max_. Subsequently a randomized sequence order was generated (MATLAB R2016b, Mathworks, Natick, MA, United States) and administered manually to the subjects starting from 2 mA below H-wave threshold up to 2 mA above M_max_ in steps of 0.5 mA. To control for post-activation depression (PAD) during H-reflex measurements an inter-stimulus interval of 10 s or longer is recommended (Crone and Nielsen, 1989; Aymard et al., 2000). However, due to the very reliable influence of PAD on the peak-to peak amplitudes of the H-reflex it seems possible to measure recruitment curves with inter-stimulus-intervals of 3 s (Zehr, 2002). As a trade-off we delivered five stimuli at each intensity with an inter-stimulus interval of 5 s to limit the effects of PAD. Due to the possible effects of head position on the size of the H-reflex (Schieppati, 1987), subjects were instructed to avoid head movement during the experiment.

### Skinfold Measurements

Skinfold thickness was measured with a skinfold caliper (Harpenden, Baty International, Burgess Hill, United Kingdom). The sum of six skinfold thicknesses over triceps, subscapular, supraspinale, abdominal, front upper thigh, and medial calf was taken to calculate body fat percentage by the equation of ISAK (Norton and Olds, 1996). Each site was measured twice. A third measurement was performed when the first two measurements deviated by more than 5%. The average from two or three measurements were taken as skinfold thickness. All skinfold thickness measurements in this study were conducted by the same experimenter.

### Cardiorespiratory Exercise Test

A cardiorespiratory exercise test was conducted on a cycle ergometer (Cyclus2, rbm elektronik-automation GmbH, Leipzig, Germany) to determine peak oxygen uptake (VO_2peak_) with an online metabolic cart (Ergostik, Geratherm Respiratory GmbH, Bad Kissingen, Germany).

The cycling protocol consisted of a warmup of 5 min at a workload corresponding to 1.5 W per kg bodyweight. An incremental ramp test proceeded the warmup. The power increment was calculated as one-third of the subject’s body weight in W to ensure similar increments in physical and psychological strain and small variations in test duration within each group. The test was terminated when cadence dropped below 65 rpm for more than 10 s or when the subjects stopped pedaling voluntarily. Every subject received strong verbal encouragement until the end of the test when surpassing a RER of 1.0 (indicative of a predominantly anaerobic metabolic state and very high intensity). Lactate probes (Lactate Pro II, Arkray, Kyoto, Japan) were obtained from the earlobe ∼5 s after test termination.

Ergometer power and heart rate was measured continuously (Polar V800, Polar Electro Oy, Kempele, Finland) and respiratory gasses were sampled breath-by-breath. All data were monitored online and stored on a computer for *a posteriori* analysis. Before each test day the gas analyzers were calibrated using ambient air and gasses of known concentration (O_2_: 15.00%; CO_2_: 5.01%). Ventilatory volumes were calibrated before each test using a 3-liter syringe.

### Data Analysis

An average of five raw EMG waveforms at each stimulation intensity was generated using Signal (v5.0.8, Cambridge Electronic Design Limited, Cambridge, England). From this average the peak-to-peak amplitudes of the H- and M-waves were used to obtain the recruitment curves as well as H_max_ and M_max_ amplitude. For the recruitment curves the averaged electromyogram amplitude was plotted against stimulation intensity which was presented as the average intensity level from the five stimulations registered by the ammeter.

**FIGURE 1:**
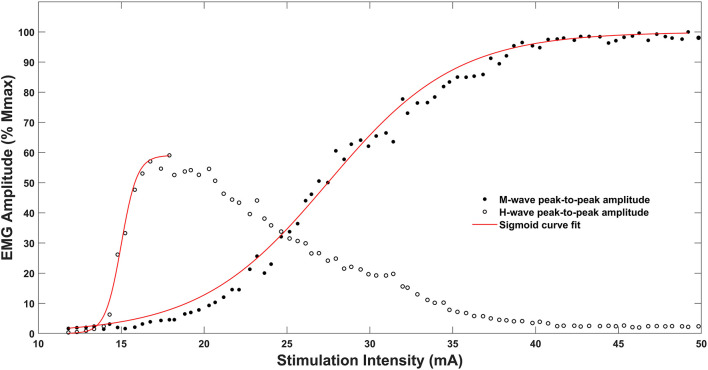
Recruitment curves and sigmoidal curve fitting for M-wave and H-reflex of the soleus muscle of one representative subject. Note that the sigmoidal fitting line for the recruitment curves stop at their peaks as the fitting procedure obtains values only until H_max_ or M_*max*_, respectively.

The slopes of the H and M recruitment curves were calculated by a sigmoid function in MATLAB (R2016b, MathWorks, Natick, MA, United States), which is recommended by Klimstra and Zehr (2008) as a preferred model for fitting the ascending limb of the H-reflex recruitment curve. A general least square model from a three-parameter sigmoidal function was implemented to fit the H and M recruitment curves (see Figure 1). The slope of the ascending limb of each recruitment curve was obtained at 50% of H_max_ and M_max_ amplitude. The equations for fitting the H and M-slopes were as follows:


$$H\,\left(s\right)=\frac{H_{\text{max}}}{1+e^{m\,\left(s\,50-s\right)}}$$

$$M\,\left(s\right)=\frac{M_{\text{max}}}{1+e^{m\,\left(s\,50-s\right)}}$$

where *H(s)* and *M(s)* is the H- or M-reflex peak to peak amplitude at a given stimulation intensity value *s*. *H*_max_ and *M*_max_ represent the upper limit of the curve, *m* is the slope parameter of the function, *s50* is the stimulus intensity at 50% peak to peak amplitude of the H_max_ or M_max_, respectively (Klimstra and Zehr, 2008). Please note that we derived H_max_ and M_max_ directly by the experimental data as this procedure yielded smaller minimal errors of the least squares model, thus improving the fit of the function for the recruitment curve.

Respiratory gasses and power data collected during the cardiorespiratory exercise tests were resampled to 1 s intervals applying a linear interpolation method in MATLAB (R2016b, Mathworks, Natick, MA, United States). Subsequently a smoothing operation was applied by applying a standard Gaussian filter (kernel: ($\sigma \sqrt{2\,\pi }){}^{-1}\text{exp}(-0.5t/\sigma^{2}$) with σ = 20 s for respiratory and σ = 3 s for power data). Single peak values from the different variables obtained after these procedures were determined as peak oxygen uptake (VO_2peak_) and maximal power (P_peak_).

### Statistics

To estimate the amount of subjects we conducted *a priori* power analyses from the study of Maffiuletti et al. (2001) from which we obtained an estimated sample size of eight subjects per group. Statistical analysis was performed using JASP (Version 0.9.2). Checks for normality were performed using the Shapiro–Wilks test. In case of a deviation from normality we applied the Mann–Whitney-U test. Levene’s test was applied to the data to check for inequality of variance. In case the assumption of equality was violated we applied the Welch test. In case of normal distribution and equality of variance of the data sets, we used independent samples Student’s *t*-test. The level of significance was set at *p* < 0.05.

## Results

### Subject Characteristics

The cyclists had an average cycling experience of 9.6 ± 5.6 years with a self-reported annual training volume of 13,000 ± 3,300 km (∼8–9 h per week). Controls were not engaged in any kind of endurance exercise nor any structured training plan but exercised irregularly in a variety of sports for 3.2 ± 3.4 h per week.

Several variables of the cardiorespiratory exercise test were highly significant between both groups, with the cyclists reaching higher values in peak oxygen consumption, peak power output (expressed in either absolute or relative values), and time to exhaustion, when compared to the controls. There were no significant effects on peak heart rate, peak lactate values, and peak RER. The skinfold measurements revealed no significant difference in body fat percentage. The mentioned physiological results are presented in Table 1.

**TABLE 1:** Results from the cardiorespiratory exercise test, skinfold caliper measurements, and MVC force recordings for cyclists and controls.

	Cyclists	Controls	*p*	*t*
VO_2peak_ (l/min)	4.62 ± 0.46	3.21 ± 0.50	<0.001	6.884
VO_2peak_ (ml/min/kg)	60.6 ± 5.79	46.3 ± 5.76	<0.001	5.793
P_peak_ (W)	430 ± 37	294 ± 40	<0.001	8.154
P_peak_ (W/kg)	5.63 ± 0.39	4.25 ± 0.41	<0.001	8.042
TTE (mm:ss)	12:40 ± 1:11	8:28 ± 1:16	<0.001	8.042
HR_peak_	192 ± 8	190 ± 10	0.581	0.561
Lac_peak_	11.3 ± 3.4	11.0 ± 3.8	0.842	0.202
RER_peak_	1.25 ± 0.05	1.29 ± 0.07	0.103	1.021
Body fat (%)	12.9 ± 2.7	14.6 ± 3.7	0.222	1.260

*Cyclists had a higher aerobic capacity, a higher maximal power and a longer time to exhaustion than controls. Values are presented as mean ± SD. VO_2max_, maximal oxygen uptake; P_peak_, Peak power output; TTE, time to exhaustion; HR_peak_, Peak heart rate; Lac_peak_, Peak lactate; and RER_peak_, Peak respiratory exchange ratio.*

### H/M Recruitment Curves

A total number of 22 participants were analyzed for soleus H/M recruitment curves. One subject from each group was removed from the analysis because a clear and distinct biphasic H-reflex could not be established reliably throughout the whole experimental session. Therefore, a total of 20 subjects (11 cyclists, 9 controls) were included into the (statistical) analysis. There was no significant difference of H_max_/M_max_ between both groups (see Table 2).

**TABLE 2:** Results for H/M recruitment curves after sigmoidal curve fitting for the cyclists and control group.

Soleus	Cyclists	Controls	*p*	*t*	Cohen’s d
M_max_ (mV)	5.13 ± 1.60	5.24 ± 2.11			
H_max_ (mV)	3.11 ± 1.22	3.20 ± 1.58			
H_max_ (% M_max_)	61.3 ± 16.6	60.0 ± 8.9	0.840	0.205	0.092
Stim. Int. H_max_ (mA)	23.0 ± 11.5	20.4 ± 9.1			
Stim. Int. M_max_ (mA)	55.2 ± 20.2	50.9 ± 14.9			
Slope M-wave (°)	76.2 ± 3.8	72.0 ± 4.4			
Slope H-reflex (°)	84.6 ± 3.0	85.0 ± 2.8			
H-slope (% M-slope)	111 ± 4.0	118 ± 6.0			

*Values presented as mean ± SD.*

*Stim. int. M_max_, Stimulation intensity at M_max_; Stim. Int. H_max_, Stimulation intensity at H_max_. Slope M-wave and slope H-reflex represent the maximal inclines derived from the sigmoidal fit.*

For the sigmoidal curve fitting procedure, we had to exclude one further subject from each group (resulting in a total of 18 subjects) because the variability of M-wave peak to peak amplitudes in the ascending limb of the M recruitment curve was too high, resulting in a fit below *r*^2^ = 0.7. The average *r*^2^ for the M- and H-wave sigmoidal fitting procedure was 0.977 ± 0.014 and 0.921 ± 0.067, respectively. Statistical analysis from the sigmoidal curve fitting data revealed a significant steeper M-slope in cyclists than in the controls. H-slope and all other parameters were not different between groups. H-slope/M-slope was significantly higher in controls than in the group of cyclists. Results for the H/M recruitment curves and sigmoidal curve fitting are presented in Table 2.

## Discussion

In the present study we investigated the H-reflex for the soleus muscle between endurance trained cyclists and controls. To the best of our knowledge this is the first cross-sectional study on this topic that tested a homogeneous group of endurance athletes characterized by a significantly higher VO2_peak_ than the control group. We found no statistical difference in the H_max_/M_max_ ratio between both groups. These results indicate that long-term endurance training might not induce higher H-reflexes as indicated by earlier studies.

### H_max_/M_max_ Ratio

**FIGURE 2:**
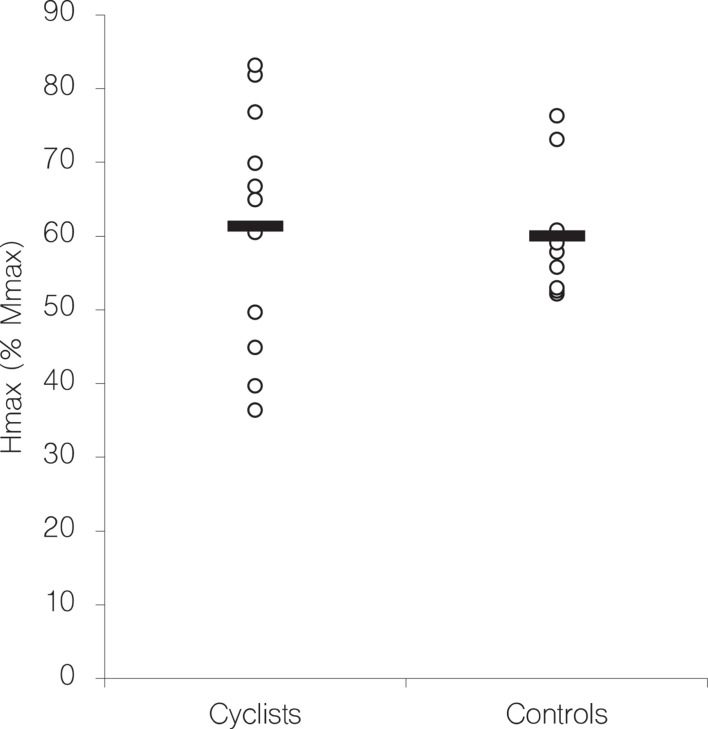
Individual H_max_ values, expressed in % of M_max_ (open circles) of the soleus H-reflex separated by group. Black bars indicate the group average.

The size of the H-reflex in the soleus muscle was not significantly different between endurance trained cyclists and controls (see Figure 2). This observation contrasts other cross-sectional studies that found higher H-reflexes in endurance trained subjects and conclude that H-reflex size seems to be related to the type of physical training (Nielsen et al., 1993; Maffiuletti et al., 2001).

To determine why this study shows opposing results, we begin with a comparative evaluation of the H_max_/M_max_ ratios in the respective studies. In this study, for the control group containing recreationally active subjects an H_max_/M_max_ ratio of 60% was obtained, with other studies showing coinciding results. Behrens et al. (2015) reveal an H_max_/M_max_ ratio of 57 and 58% for two groups of comparable activity levels, while Nielsen et al. (1993) have found an average H_max_/M_max_ ratio of 62% in a comparable group. A smaller ratio of 52% is presented by Perot et al. (1991) that can be explained by a less comparable group including both recreationally trained and sedentary subjects, the latter probably contributing to the smaller ratio, as in these subjects smaller H_max_/M_max_ ratios are manifested in general (Nielsen et al., 1993; Maffiuletti et al., 2001). When comparing the H_max_/M_max_ ratios of endurance athletes there is less consensus and altogether very limited data. While Ogawa et al. (2009) present a H_max_/M_max_ ratio in the soleus muscle of 72% for a group of swimmers, Maffiuletti et al. (2001) determined a ratio of 67% for a mixed group of athletes trained in several endurance sports (triathletes, swimmers, and cross-country skiers). Although not substantially lower, the H_max_/M_max_ ratio of only 61% in the endurance-trained group in this study is probably a crucial factor for the contrasting results to the aforementioned studies. However, it has to be acknowledged that methodological differences in the experimental protocols of the studies referenced above might limit the direct comparison of H_max_/M_max_ ratios. In this regard it has been shown that the use of single bipolar EMG electrodes does not account for spatial variations of compound muscle action potentials across large muscles (Vieira et al., 2015). In consequence, H_max_/M_max_ ratios could differ significantly for those studies with the EMG electrodes positioned on different sites of the soleus muscle.

A more manifest reason why the H_max_/M_max_ ratio in the endurance group is lower in this study, might be that the subjects in this study have a different sporting history. This study included only cyclists, whilst other studies have not incorporated any cyclists but athletes from various endurance sports. Therefore, one could assume that the different types of motor tasks could have an effect in specific spinal adaptations in the Ia spinal reflex pathway. As this is the first study to investigate H-reflex parameters in cyclists, in fact it is the only study incorporating subjects from only one type of endurance sport.

One such specific spinal adaptation might be a modification in the transmission of the Ia-volley at the afference/α-motoneuron synapse (Nielsen et al., 1993; Meunier et al., 2007; Vila-Cha et al., 2012). These kinds of adaptations could occur from a purely neural side without any changes in fiber type content. Nielsen et al. (1993) state that presynaptic inhibition in non-endurance trained athletes could lead to a lower transmission across the synapses resulting in a smaller H-reflex. Conversely this would mean that endurance trained athletes in their group display less presynaptic inhibition, leading to a higher transmission of the Ia-Volley across the synapse, consequently leading to a higher H-reflex. Since, we could not find a higher H-reflex in the group of cyclists, this assumption implicates that cyclists may not show an adaptational response toward less presynaptic inhibition compared to other endurance athletes.

### Parameter Fitting of Recruitment Curves

Additionally, to the H_max_/M_max_ ratio, slope parameters of the H and M recruitment curves were analyzed to potentially gain further insights into motor unit recruitment characteristics of well-trained endurance cyclists. A steeper slope of the H recruitment curve represents a higher “reflex gain,” defined as an increase in the number of recruited motor units relative to the increase in stimulation intensity of the Ia afferent nerve fibers (Funase et al., 1994). Therefore, a sigmoidal fitting procedure for the slopes of the H and M recruitment curves was implemented and statistically evaluated (see Figure 1). We could not show any differences between groups in the slope parameters of the H recruitment curve. However, we revealed a slightly significant (*p* = 0.046) steeper slope of the M recruitment curve in the group of cyclists. Very likely associated to this finding, we also demonstrate a significantly reduced H-slope/M-slope ratio in the group of endurance athletes.

The steepness of the slope of the M recruitment curve is said to be indicative of the level of muscle homogeneity as it depends on the distribution of motor axons in relation to their diameter (Piscione et al., 2012). The steeper M-slope in the soleus muscle in our results suggests a narrower distribution of motor axon diameters in the group of cyclists than in the control group, representative of a higher content in slow motor units. This finding coincides with the well acknowledged muscle fiber type shift toward type-I muscle fibers as a result of long-term endurance training, supporting the assumption that this parameter can be taken as a (non-invasive) marker of muscle homogeneity.

The H-slope/M-slope ratio is assumed to give a more sensitive estimation of motoneuron excitability than the H_max_/M_max_ ratio (Funase et al., 1994; Piscione et al., 2012). We have shown a significantly reduced H-slope/M-slope ratio in the group of cyclists. This is possibly attributed to differences in the M-slope in the group of cyclists, who show greater mean slope values than the control group, whilst H-slope mean values are similar between groups. It is therefore debatable whether a different H-slope/M-slope ratio is representative of a higher motoneuron excitability. It is rather the H-slope that should be modified to indicate alterations in motoneuron excitability.

## Limitations

We measured H-reflexes during rest in supine position which might be considered as a second limitation of the present study. It is recommended to measure H-reflexes for SOL in a seated position and during a background muscle contraction of 5–10% MVC (Pierrot-Deseilligny and Burke, 2005). In the present study, however, we opted for a supine position and resting condition for the following reasons. A rest condition was chosen because our primary aim was to test the hypothesis that H-reflexes are generally higher in endurance trained athletes and thus we decided to avoid any contraction of the respective muscle which might induce a sports specific modulation of the H-reflex. Moreover, as we were not able to control for possible modulations from spinal inhibitory interneuronal circuits like presynaptic, reciprocal, or recurrent inhibition that are known to influence the size of the H-reflex amplitude (Schieppati, 1987; Pierrot-Deseilligny and Mazevet, 2000; Zehr, 2002; Misiaszek, 2003; Knikou, 2008), we have followed the recommendations by Zehr (2002), who suggested that the lowest influence of the above mentioned neural mechanisms is in a supine position. However, this approach complicates the comparison to other studies that have measured H-reflexes in other positions.

## Conclusion

The results of this study indicate that well-trained cyclists are possibly not affected by use-dependent plasticity in the spinal cord in regard to an increased H-reflex. Despite methodological differences to other studies showing spinal adaptations for endurance athletes, we suggest that the training-specific control of the endurance sport itself could be of importance in the modulation of the H-reflex pathway. We assume that presynaptic inhibition could be the relevant selective adaptational response to a specific endurance training, with less presynaptic inhibition in tasks like running or cross-country skiing, and more presynaptic inhibition in tasks like cycling.

The significantly steeper H-slope/M-slope ratio in the control group indicates toward a higher motoneuron excitability for this group. However, this ratio is expected to rise with a steeper slope of the H-reflex recruitment curve, which is not the case in this study. Moreover, the higher ratio is obtained by a significantly flatter slope in the M recruitment curve in the group of cyclists, raising doubts whether this phenomenon is indicative of a higher motoneuron excitability in the controls.

Recent findings suggests that more emphasis should be made toward understanding neural processes and adaptations occurring at the spinal level as a cause of long-term physical activity, which is far from understood. Deeper knowledge in this area could potentially show that the CNS plays a more significant role in athletic performance than we currently assume.

## Data Availability Statement

The raw data supporting the conclusions of this article will be made available by the authors without undue reservation.

## Ethics Statement

The studies involving human participants were reviewed and approved by the Ethics Committee of the University Konstanz. The patients/participants provided their written informed consent to participate in this study.

## Author Contributions

RB devised the project, designed the study, carries out the experiments, performed the computations and data analysis, and wrote the manuscript. L-SG reviewed the manuscript and helped with the data analysis. MG reviewed the manuscript. All authors contributed to the article and approved the submitted version.

## Conflict of Interest

The authors declare that the research was conducted in the absence of any commercial or financial relationships that could be construed as a potential conflict of interest.

## Publisher’s Note

All claims expressed in this article are solely those of the authors and do not necessarily represent those of their affiliated organizations, or those of the publisher, the editors and the reviewers. Any product that may be evaluated in this article, or claim that may be made by its manufacturer, is not guaranteed or endorsed by the publisher.
